# Game-Based Dual-Task Exercise Program for Children with Cerebral Palsy: Blending Balance, Visuomotor and Cognitive Training: Feasibility Randomized Control Trial

**DOI:** 10.3390/s22030761

**Published:** 2022-01-19

**Authors:** Tony Szturm, Sanjay Tejraj Parmar, Kavisha Mehta, Deepthi R. Shetty, Anuprita Kanitkar, Rasit Eskicioglu, Neha Gaonkar

**Affiliations:** 1 College of Rehabilitation Sciences, University of Manitoba, Winnipeg, MB R3E0T6, Canada; kavisha.mehta1305@gmail.com; 2 SDM College of Physiotherapy, Dharwad 580009, India; vani.ravishetty@gmail.com; 3 Department of Applied Health Sciences, University of Manitoba, Winnipeg, MB R3E0T6, Canada; anuprita.kan@gmail.com; 4 Computer Sciences, University of Manitoba, Winnipeg, MB R3E0T6, Canada; Rasit.Eskicioglu@cs.umanitoba.ca; 5 Pediatric PT, KSS School, Valpoi 403506, India; Nehagaokar@gmail.com

**Keywords:** cerebral palsy, balance training, telerehabilitation, dual-task training

## Abstract

The objective of this exploratory randomized controlled trial (RCT) was to provide evidence for the feasibility and therapeutic value of a novel game-based dual-task balance exercise program in children with cerebral palsy (CP). Twenty children with CP were recruited and randomized into two groups: (a) the conventional balance training group (CG) and (b) the experimental group (XG), which received a game-based dual-task (DT) balance exercise program. Both groups received their respective therapy programs for 12 weeks at a frequency of three sessions per week. Semi-structured interviews with the parents and children and qualitative analysis were conducted to evaluate the children’s experiences with the game-based exercise program. The quantitative analysis included (a) the Pediatric Balance Scale (PBS), (b) Gross Motor Function Measure-88 (GMFM-88), and (c) computerized measures of standing balance performance during various dual-task conditions. Compliance was 100% for all 20 participants. Four themes captured the range of each participant’s experiences and opinions: (a) reasons for participation, (b) likes and dislikes with the technologies, (c) positive effects of the program, and (d) future expectations. Children in the XG demonstrated greater improvements in PBS, GMFM, and DT balance measures as compared to children in the CG. The findings demonstrate feasible trial procedures and acceptable DT-oriented training with a high compliance rate and positive outcomes. These findings support further research and development and progression to the next phase of a full-scale RCT to evaluate the clinical effectiveness of the game-based DT balance exercise program for children with CP.

## 1. Introduction

Cerebral palsy (CP) is one of the most common motor neurodevelopmental disorders, which affects five of every thousand live births in India and three of every thousand live births in North America [1,2]. Balance impairments and mobility limitations are common in children with CP [3,4,5]. Due to the many problems associated with reduced balance control, providing an effective rehabilitation program is essential in the development of the child’s mobility skills and in helping to prevent non-use sequelae, such as bone deformities, contractures, and obesity [6,7]. Clinicians and researchers have established the importance of task-specific repetitive training in the rehabilitation of motor function in children with cerebral palsy [7,8]. If improved balance is an expected outcome, training in tasks involving the facilitation of balance reactions in standing would be beneficial [9].

Balance is a functional term, and its control is a complex multidimensional process.

Independent community walking requires adequate balance skills and cognitive flexibility to manage a variety of environmental demands, which include attending to and interacting with various objects, searching for information, processing what is being seen, reading, etc. Balance and cognitive abilities are closely linked, and several studies have demonstrated dual-task interference with balance in children with CP when performing a concurrent cognitive task [5,6,7,8,9]. Therefore, DT training programs that use graded balance challenges combined with information processing loads would enhance rehabilitation outcomes.

An emerging approach to increase compliance is to combine balance exercises with computer games, making training a more engaging experience [10,11,12,13]. Several gaming systems have been used in the rehabilitation of balance in children with CP, which include Nintendo Wii Fit [14,15,16], Microsoft Xbox Kinect [16,17], Dance Mat [18], and PlayStation Eye Toy [19]. Other purpose-built rehabilitation gaming systems include various force platforms/pressure mats, which record the center of foot pressure signals (COPs), or video cameras to interact with computer games [20,21,22].

Game-based rehabilitation approaches have the potential to improve clinical outcomes and enhance the active participation of children with neuromotor deficits [12,23,24]. However, there are only a small number of exercise games available for either commercial gaming systems or custom exergaming systems. Therefore, there is a limited selection of cognitive activities, whereas there is a large number of inexpensive and readily available common and modern commercial computer games that are engaging, are therapeutic, and have a broad range of cognitive activities that can be played with a computer mouse or equivalent [25,26]. Based on this limitation, a low-cost, engaging dual-task computer game-based rehabilitation system (GRS) was developed for an integrated approach to balance, visuomotor, and executive cognitive training appropriate for children with CP. It uses an inertial-based (IB) mouse, a novel hands-free plug-and-play computer game controller, to interact with cognitive computer games while performing a wide range of balance exercises [27,28,29,30,31]. The IB mouse functions as a responsive USB plug-and-play computer mouse, and this allows common and modern computer games to be used and enjoyed as part of the rehabilitation program. Therefore, therapists and children can choose from a large variety of existing common and modern computer games. Many inexpensive, commercially available games involve multitasking. Hence, the tasks also engage key attentional, perceptual, and executive cognitive skills.

The purpose of this exploratory randomized clinical trial (RCT) is to provide evidence for the feasibility of conducting a full-scale RCT using the GRS for DT gait training in CP children with balance impairments. The first objective was to explore the implementation, acceptability, and appropriateness of the game-based DT balance program. Semi-structured interviews were conducted to explore children’s parents’ experiences with their respective exercise programs. The qualitative findings of participants’ experiences help to identify (a) perceived exercise benefits, (b) difficulties with the exercises and using the technologies, and (c) the engagement and motivational value of the computer games. The secondary objective was to estimate the treatment effect size of the DT balance program compared to usual physiotherapy. The working hypothesis was that the group receiving the DT balance program would demonstrate significantly greater improvement in balance, gait performance, and cognitive measures compared to usual physiotherapy.

## 2. Methods

**Figure 1:**
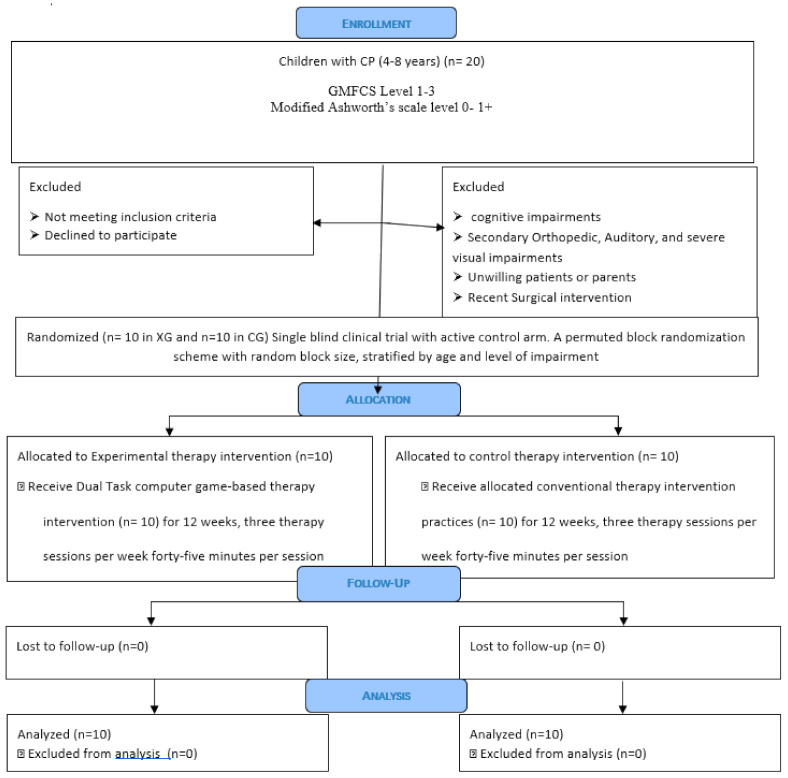
CONSORT Flow Diagram.

Children diagnosed with CP were recruited for this single-blind randomized clinical trial with an active control arm (see Figure 1 for CONSORT diagram). The study was conducted according to international standards of Good Clinical Practice. Ethics approval was obtained from the Health Research Ethics Board (HREB), University of Manitoba, Winnipeg, Canada, and the Institutional Ethical Committee of Shree Dharmasthala Manjunatheshwara (SDM). University, Karnataka, India. This study is registered at ClinicalTrial.gov with the identifier # NCT03873441. The study participants were recruited from the Pediatric Physiotherapy Outpatient Department (OPD) of SDM College of Medical Sciences and Hospital. Successfully screened participants were randomized to the experimental group (XG) or control group (CG) by having them choose one of 20 sealed opaque envelopes that contained a letter signifying the group assignment. A graduate student not involved in the study produced the envelopes: 10 that contained a letter for the experimental group and 10 for the control group. The envelopes were thoroughly shuffled before each selection.

Inclusion criteria: (a) children with a confirmed medical diagnosis of CP, ages 4–8 years, (b) Gross Motor Function Classification System level 1–3 [32], and (c) Modified Ashworth Scale level 0 to +1 [33]. Exclusion criteria: (a) visual or auditory impairment preventing them from seeing and interacting with the video games, (b) secondary orthopedic complications, (c) recent surgical intervention, (d) cognitive impairment, (e) seizures, or (f) complex communication disorders.

## 3. Procedures, Tests, and Instrumentation

The following outcome measures were obtained pre- and post-intervention by a physiotherapist masked to the intervention assignment.

**Figure 2:**
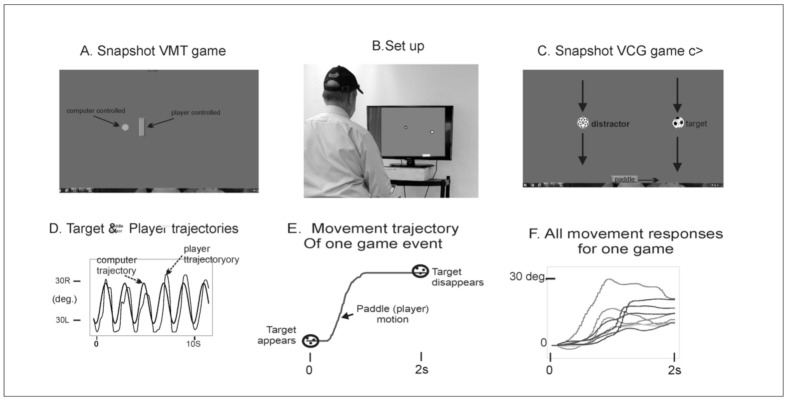
Illustration of the assessment game setup. As presented in Panel (**B**), the participant stands while viewing a computer monitor. An inertial-based mouse is secured to the sports cap, and head rotation is used to interact with the visuospatial cognitive games. Panels (**A**,**C**) present snapshots of the VMT and VCG modules. Panel (**A**) (VMT) shows cursors of different shapes appearing on the computer monitor. The target is a circle, and its motion was computer-controlled and moved at a predetermined frequency of 0.5 Hz with an amplitude of 70% of the monitor width. The second cursor is a rectangle, which is slaved to the head-mounted IB mouse. Children were instructed to rotate their heads and overlap the rectangle cursor with the target circle for several cycles (i.e., 30 s). The coordinates of the rectangle and target cursor were recorded at 100 Hz for offline analysis. Panel (**D**) presents synchronous plots of the target cursor motion and user movement trajectories (head rotation) for a typical VM tracking task. The maximum is the leftmost position, and the minimum is the right-most position. Panel (**C**) (VCG) objects are categorized as targets or distractors. The game objects appear at random locations at the top of the display every 2 s and move in a diagonal trajectory to the bottom of the display. Children were required to move the paddle to catch the target objects while avoiding the distractors. The coordinates of the game paddle and target objects were recorded (100 Hz) for offline analysis of the success rate (SR). Panel (**E**) presents the trajectory for one VCG game movement response from target appearance to target disappearance. Panel (**F**) presents overlay trajectories of all game movement responses for one game session.

1. Pediatric Balance Scale (PBS): It consists of 14 items, which include: standing unsupported, standing with a narrow base of support (BOS), tandem standing, turning, and forward reach [34].
2. Gross Motor Function Measure-88 (GMFM-88): The GMFM-88 is an observational clinical tool designed to evaluate a change in gross motor function in children with CP aged between 5 months and 16 years. The standing and the walking, running, and jumping subtests were used [35].
3. Modified Clinical Test of Sensory Integration in Balance (MCTSIB) and computerized dual-task (DT) balance assessment: This assessment included four tasks that were first performed on a fixed floor surface and then on a compliant sponge pad. The children were asked to stand still for a duration of 30 s with eyes open (EO) or with eyes closed (EC) while performing a visuomotor tracking task and a visual cognitive game task described by Bhatt et al., (2019) 31] and Szturm et al., (2015) [36]. The VMT module, as described in Figure 2A,B, involved tracking a visual target that moved horizontally on a computer display for several cycles. As presented in Panel B, the participant stands while viewing a computer monitor. An inertial-based mouse is secured to the sports cap, and head rotation is used to interact with the visuospatial cognitive games. Panels A and C present snapshots of the VMT and VCG modules. Panel A (VMT) shows cursors of different shapes appearing on the computer monitor. The target is a circle, and its motion was computer-controlled and moved at a predetermined frequency of 0.5 Hz with an amplitude of 70% of the monitor width. The second cursor is a rectangle, which is slaved to the head-mounted IB mouse. Children were instructed to rotate their heads and overlap the rectangle cursor with the target circle for several cycles (i.e., 30 s). The coordinates of the rectangle and target cursor were recorded at 100 Hz for offline analysis. Panel D presents synchronous plots of the target cursor motion and user movement trajectory (head rotation) for a typical VM tracking task. The maximum is the leftmost position, and the minimum is the right-most position. Panel C (VCG) objects are categorized as targets or distractors. The game objects appear at random locations at the top of the display every 2 s and move in a diagonal trajectory to the bottom of the display. Children were required to move the paddle to catch the target objects while avoiding the distractors. The coordinates of the game paddle and target objects were recorded (100 Hz) for offline analysis of the success rate (SR). Panel E presents the trajectory for one VCG game movement response from target appearance to target disappearance. Panel F presents overlay trajectories of all game movement responses for one game session.

Prior to testing, the participants were allowed to play the tracking and game tasks while sitting for a few minutes to become familiar with each task.

The displacement of the center of foot pressure (COP) was recorded using a flexible force sensor array (FSA) mat (Vista Medical, sampling frequency 80 Hz). The FSA pressure mat was placed on top of the sponge to record the instantaneous COP position during each task condition [31,36,37,38].

## 4. Interventions

Two therapists delivered the therapy protocols, one to the XG and one to the CG. Each group received the protocol for 12 weeks at a frequency of three therapy sessions per week. Each session was 45 min long.

The control group (CG) received a conventional physical therapy balance program.

This included (a) active-assisted stretching exercises and (b) balance and weight-shifting exercises with arm reaching and trunk bending movements. These activities were initially performed on a fixed surface, gradually progressing to unstable surfaces. As tolerated, the balance challenge was progressed by increasing the thickness of the sponge pads and using air-bladder-type balance disks and bolsters.

The experimental group (XG) received the game-based DT balance training program.

Children were fitted with a headband/cap instrumented with the miniature IB mouse to play various cognitive computer video games. The children were asked to stand and balance on an unstable surface (as tolerated) while playing various computer video games. As with the CG, the balance challenge was progressed by increasing the thickness of the sponge pads and then using balance disks and bolsters. The amount of cognitive demand was graded by selecting different computer video games and adjusting the difficulty level of the games. Many real-life tasks involve head movements to search and track various objects, avoid distractors, and process information on what is being seen. Appendix A presents a list of computer games used in the present study.

## 5. Qualitative Analysis

The qualitative analysis, i.e., post-intervention interviews, was conducted only on the experimental group to explore the experiences of the children using the gaming technology and game-based DT exercise program. The interview focused on generating information on experiences and the views of the parents and children about the expectations, likes and dislikes, and outcomes of using the balance gaming exercise program. The following questions were asked during the interview.

1. When you agreed to participate, how did you and your child hope to benefit from the therapy program?
2. Were there things about the game or exercise program that you liked and things you did not like?
3. What did you think about the computer games your child was asked to play? Did your child enjoy the games? Were there games that you did not seem to enjoy?
4. Did you feel that this therapy program helped?
5. If you were provided with the right setting, would you want to continue with these game-based exercises?

A research assistant who was blinded to the interventions conducted the interviews. A second person was present to record all responses in writing. The analytical framework of interpretive description was used for thematic interpretation. One researcher who developed the coding system by paraphrasing, generalizing, and abstracting the written responses of each interview initially read the written response transcripts. A second researcher scrutinized the coded data and identified any additional unique responses. The two researchers then met, and a final coded response category was produced and organized into final themes [39].

## 6. Quantitative Analysis

The following outcome measures were obtained pre- and post-intervention:

1. Balance performance: The total path length (TPL) of COP excursion over the 30 s task duration was computed [31]. A decrease in TPL was interpreted as improved balance performance.
2. Visuomotor (VMT) performance measure: Synchronous plots of the target motion and user’s head rotation (rectangle cursor) for a typical VMT task are presented in Figure 2. The total residual error (TRE) was determined by computing the difference between the trajectories of the target and head cursor motions and expressed as a percentage of display width [31,36].
3. Visual cognitive game (VCG) performance measure: Figure 2 depicts the overlay trajectories of game paddle displacements (head movements) for all game events in one 60 s game session. Each game event lasted for two seconds from initial target appearance to target disappearance. The success rate (SR)—the percentage of targets caught—was quantified [31,36].

## 7. Statistical Analysis

Descriptive statistics, including means, standard error of mean (SEM), and percentages, were used to describe demographic variables and outcome measures. Effect size was calculated using Cohen’s d [40]. A value of 2 is a “small” effect size, 0.5 represents a “medium” effect size, and 0.7 is a “large” effect size. The normality of the data was assessed using the Shapiro–Wilks test. This test revealed a normal distribution, *p* > 0.1, for all outcome measures. A two-way repeated-measures ANOVA was used to examine the effects of time (pre- and post-intervention) in each group (XG vs. CG) and time*group interaction on balance, VMT, and VCG outcome measures. The effect size was calculated using Cohen’s d [31,36]. The significance level was set at 0.05, and statistical analysis was conducted using SPSS (version 27) (IBM SPSS Science, Chicago, IL, USA).

## 8. Results

The recruitment target of 20 children was reached in 6 months. All children screened and randomized to their respective exercise groups completed the pre- and post-intervention assessments and attended all exercise sessions (compliance of 100%). There were no adverse events or problems with the use of the technology or computer games in therapy. These findings demonstrate excellent feasibility.

Table 1 presents the demographic data. The mean age of children in the XG was 6.34 years, and for the CG, it was 6.33 years. There was no significant difference in age between the two groups (*p* = 0.3). Both groups had the same number of females and males, and both groups had similar numbers of GMFCS levels one to three.

The following four themes captured the range of parents’ and children’s experiences and parents’ opinions about their child’s exercise program. Table 2, Table 3, Table 4 and Table 5 present parents’ direct quotes for each theme.

1. Expectations of therapy (Table 2): All parents expressed their concerns regarding their child’s poor balance, limited mobility, and inability to participate in play and most school activities. Of note, all participants had been undergoing conventional therapy to improve balance for at least one year.
2. Likes and dislikes (Table 3): Most parents mentioned that balance activities on the different surfaces, especially the balance disks, were very difficult to carry out while playing the computer games and that this frustrated the children. Most parents and children reported that the chosen computer games were fun to play. One parent commented that it was easier to convince children to perform balance exercises when using computer games than conventional exercises.
3. Effects of therapy (Table 4): All parents gave positive feedback, such as improvement in balance performance, enhanced concentration, increased confidence, improved walking abilities, improvement in stair climbing, and improved functional activity.
4. Future expectations (Table 5): All parents and children expressed their interest in continuing this therapy if they were provided with the gaming system.

**Figure 3:**
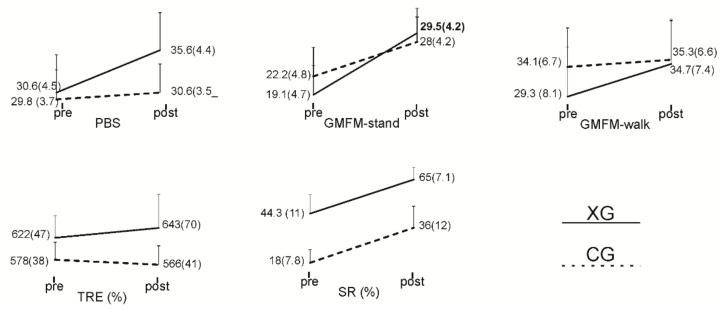
Line plots presenting group means and standard errors of means (SEM) pre- and post-intervention.

Table 6 presents the ANOVA results for time, group, and interaction effects for all outcome measures. Group means and standard errors of means (SEM) by group and time period are presented in Figure 3. A significant improvement in PBS and GMFM subscores pre- to post-intervention (time effect) was seen for both groups. Effect sizes were medium to large, ranging from 0.2 to 0.6. There were no significant time*group effects. On average, PBS increased by 5 for the XG and 0.8 for the CG, GMFM standing increased by 10.4 for the XG and 5.8 for the CG, and GMFM walking, running, and jumping increased by 5.5 for the XG and 1.2 for the CG.

In the case of COP TPL, there was no significant time effect, but there was a significant time*group effect for all task conditions except for the VCG condition. In the case of the VCG condition, there was a significant decrease in TPL for both groups, with a large effect size of 0.5. There was no time*group or group effect. The results of further statistical comparisons of time effects by group revealed a significant decrease in COP TPL pre- to post-intervention for the XG, but no significant change was observed for the CG (Table 7). The effect size for the XG results was large, ranging from 0.5 to 1.1. There was one exception in each group. There was no significant change in TPL for the eyes-closed condition in XG, while there was a significant increase in TPL post-intervention for the VCG condition in CG.

As presented in Table 6 and Figure 3, there was a significant improvement in SR pre- to post-intervention in both the XG and CG and no significant time*group effect. The effect size was moderate, with a value of 0.3. In the case of TRE, no significant effects were observed for time or time*group. 

There were no significant group effects observed for any of the outcome measures except for VCG SR, where performance was significantly better in the XG as compared to the CG.

The VCG module, as described in Figure 3, involved moving a game paddle (head rotation) to interact with moving game objects.

## 9. Discussion

The purpose of the present study was to evaluate the feasibility of conducting an RCT using a game-based DT exercise program in children with CP. The present study findings demonstrate that the parents recognized that their expectations related to improving children’s balance skills were addressed during the 12-week therapy program. Parents also shared the challenges that they faced regarding children’s participation in therapy. There are several likely reasons for the 100% compliance. Continued guidance, instructions, and assistance were provided to the children throughout the intervention to ensure that they understood how to play the games, and they were provided a variety of games with appropriate difficulty levels, as well as cognitive activities. As reported by most of the participants, the interactive commercial computer games used in the present study were engaging and fun, and most of the content was deemed appropriate.

There was little difficulty in using the technology (i.e., a wireless plug-and-play computer mouse and computer games), although it was noted by all participants that the dual-task exercise program was challenging. The acceptability of the gaming system and game-based exercise program was shown through the comments of all participants.

The present study observed a significant decline in COP excursion post-intervention for all task conditions for the XG with a medium to large effect size, ranging from 0.5 to 1.1. In contrast, there was no significant change observed in the CG. Other studies have used COP excursion to evaluate the effects of game-based balance exercise programs in CP children [41,42,43,44]. In these studies, the coordinates of the COP were used to control the motion of a game sprite to catch falling game targets. A 2.6% decrease in COP excursion post-intervention was observed when assessed while children stood on a fixed floor surface with eyes open. The present study extends these results and observed greater decreases in COP excursion when balance was assessed while standing on a sponge surface and while performing the visual tracking and cognitive game activities.

Both the XG and CG showed significant improvements post-intervention in clinical measures of balance and locomotion with medium to large effect sizes. The minimal clinically important difference (MCID) for the PBS is reported to be 3.7 for children with CP. In the present study, a greater improvement in the PBS score of 5 was observed for the XG. The MCID reported for the standing subtest of GMFM ranges from 3 to 5 for children at GMFCS levels 1–3. In the present study, greater improvements were observed for both groups: 10.4 for the XG and 5.8 for the CG. The MCID reported for the walking, running, and jumping subtest of GMFM ranges from 1.8 to 4 for children at GMFCS levels 1–3. In the present study, a greater improvement of 5.5 was observed for the XG. These improvements in PBS and GMFM are complemented by qualitative data: i.e., the parents reported a change in the children’s level of independence in their daily tasks at home, at school, and in play.

In the present study, different sponge pads and air-bladder balance disks were used during the DT exercise program to grade the balance challenge. A compliant support surface cannot reciprocate the normal forces from the feet as the body sways. The result is an increase in the frequency and amplitude of body sway movements in all directions [37,45], a condition to which the child must quickly sense and correct to minimize body sway and prevent loss of balance. The increased cognitive demand during balance activities can also result in increased body sway, i.e., a dual-task interference effect [40,45]. The computer games used in the present study require executive cognitive functions to process what is being seen, such as visual attention, visual search, and spatial processing of randomly moving game targets and distractors (cognitive inhibition). The ability to maintain and restore standing balance on a compliant surface also requires processing and organizing spatial information from multiple sensory systems, including vision, to determine body orientation and the direction and amplitude of body motion. Neuroimaging studies have shown that the prefrontal cortex is strongly activated in CP children when performing dual tasks [46,47,48,49,50]. Dual-task balance training on unstable surfaces involves more unpredictable balance disturbances as compared to that received during the CG training. Therefore, the children in the XG would be exposed to considerably more situations that require reactive balance control needed to restore balance. This is likely the reason that a significant post-intervention reduction in COP excursion was observed in children in the XG but not the CG.

Adding a gaming element was intended to provide extra motivation for the children in the form of a challenge and a more enjoyable means of encouraging them to follow repetitive movements that are a part of the rehabilitation process. Most parents commented that the combination of games and balance exercises was challenging yet engaging and that their children enjoyed playing the games. As parents mentioned, the addition of game tasks with colorful backgrounds and attractive characters enhanced their children’s concentration and improved children’s compliance with the intensive balance gaming tasks. Studies in the past comparing the use of video games to that of traditional therapy showed similar results [47,48,49].

## 10. Conclusions

The game-based DT methods presented in this study broaden the type of visuospatial cognitive activities that can be combined with balance training in children with CP. The findings demonstrate evidence on recruitment, the feasibility of trial procedures, 100% compliance, the acceptance of the game-based DT exercise program, and the usability of the gaming system (mouse and computer games). Clinically meaningful changes in balance performance were observed with moderate to large effect sizes. One limitation relates to how children spontaneously prioritize their attention between balancing and tracking/cognitive tasks during DT testing. It is possible that balance was prioritized and that the children did not fully attend to and process the cognitive information on the computer monitor. Alternatively, the information was received and processed, but the performance was affected due to dual-task interference. Modest, fair to good performance levels were observed for cognitive game tasks during testing. This demonstrates that the children were attending to and processing the information seen on the display. However, they may have stopped intermittently for a few seconds and prioritized balance processing.

The long-term effects of DT balance exercise in children with CP will need to be confirmed in future adequately powered randomized controlled trials. In addition to measures of structure and function, future randomized controlled trials should also include outcome measures such as health-related quality of life and level of participation to validate the findings. For children, outcome measures such as the Goal Attainment Scale could be used [51]. A clinical trial is also planned to extend the present application to include game-based DT treadmill training.

## Figures and Tables

**Table 1:** Demographic data.

Experiment	Mean Age (SD)	Gender	GMFCS Level
XG	6.34(2.3)	F-3 M-7	I-5 II-3 III-2
CG	6.33(2.2)	F-3 M-7	I-6 II-4 III-0

**Table 2:** Expectations and problem list.

	Expectations	Problem List
Participant	Quotes from the interview:	Quotes from the interview:
P1	“This therapy, which included both balancing as well as playing the game simultaneously would help him to become better at balance and doing challenging tasks	“He always had frequent falls while, crossing hurdles/obstacles.” “He was not confident running.”
P2	“This therapy where the balance was challenged would help her improve her independence while walking.”	“My daughter could not walk independently initially”; “She had frequent falls.”
P3	“Standing on different surface would help him in his balance. I thought this treatment would help him with his balance issues.”	“She was scared to leave my hand and walk” “He always had his knees bent while standing and walking”
P4	“Standing on different surfaces along with playing games was the therapy and I feel this would help him a lot, as it was very good thing to improve his balance”	“had frequent falls.”
P4	“Also his concentration.”	
P5	“Before participating in this program, my son did not walk independently; he was scared to do so. I believed he would do so by playing the games while standing on different platforms.”	“my son did not walk independently” “he was scared to do so.”
P6	“I thought she would finally walk confidently, thus enrolled her in the therapy.”	“My daughter while joining this therapy was always scared to walk”
P7	“Playing the games while standing on different surfaces would increase his concentration “and his balance.”	
P7	“I thought my son would finally concentrate on one thing the games while standing/balancing on the floor, surfaces (bolster, balance disk)”	

**Table 3:** Likes and dislikes.

	Likes and Dislikes	Choices of Games
Participant	Quotes from the interview:	Quotes from the interview:
P1	“There was nothing I did not like.”	“He loved Zhu Zhu Pets and Feeding frenzy”
P2	“I liked how computer games were used.”, “this was more fun than the typical therapy programs my child has done in the past.”	“She loved all the games, chicken invaders she liked the most.”
P2	“Different surfaces was challenging for her.”	
P2	“Usually she loves colorful things, so these games she enjoyed.”	
P3	“as games got difficult, he was a little uncomfortable initially. But he did get better and did improve.”	“Aditya loved playing feeding frenzy, birds town, zhu zhu pets”
P3	“The games got challenging as well, which he really enjoyed.”	
P4	“When the games got difficult, it was challenging for him to balance and this was frustrating With practice he got better.”	“Zhu zhu pets, feeding frenzy he enjoyed.”
P5	“He loved all the games enjoyed watching the cartoons and playing the colorful games.”	“Feeding frenzy, birds’ town he enjoyed.”
P6	“She loved all the games. Loved colorful cartoons and thus was interested and played mindfully.”	“She loved all the games.”
P7	“He loved colorful cartoons on the screen.”	“The concentrated on the screen, loved playing chicken invaders and birds town.”

**Table 4:** Effects of the game-based exercise program.

Participant	Quotes from the Interview:
P1	“Different surfaces on which he stood improved his balance I feel”
P1	“His concentration improved.”
P1	“Yes he now crosses hurdles, climbs stairs without losing his balance.”
P1	
P2	“She now is less fearful to walk.”
P2	“Her activities like climbing on the bed/chair has begun.”
P3	“His concentration also improved.”
P3	“Yes his knees are now more straight, and he is confident while walking, stair climbing, crossing hurdles.”
P3	
P3	
P4	“Yes his balance has surely improved and is more confident.”
P4	
P5	“Yes my son now walks independently after the 12 weeks session.”
P6	“As she moves her head while playing the games, her balance was challenged. This helped her improve her control over her balance and feet.”
P6	
P7	“His concentration has increased”
P7	“Now stands and walks with minimal falls.”
P7	“Now stands and walks with minimal falls.”

**Table 5:** Future expectations.

Participant	Quotes from the Interview:
P1	“Yes I would like to continue the therapy.”
P2	“Yes, I would love to continue this therapy as it has worked well for my daughter.”
P3	“Yes”
P4	“Yes”
P5	“Yes”
P6	“Yes”
P7	“Yes, I would love to continue the therapy.”

**Table 6:** Presented ANOVA results are f-statistic (f), *p*-value (*p*), and effect size (ef) for each outcome measure. All conditions, eyes open (EO), eyes closed (EC), VCG, and VMT, were performed while standing on a sponge.

	Time (f, *p*, ef)	Group (f, *p*, ef)	Time*Group (f, *p*, ef)
TPL-EO	0.7, 0.4, 0.04	0.2, 0.7, 0.01	6.5, 0.02, 0.3
TPL-EC	1, 0.3, 0.1	2.4, 1.1, 0.1	4.9, 0.04, 0.2
TPL-VMT	2, 0.2, 0.1	3.1, 0.1, 0.2	5.7, 0.03, 0.2
TPL-VCG	20.6, 0, 0.5	0.5, 0.5, 0.03	0.9, 0.3, 0.1
VCG-SR	8, 0.01, 0.3	5, 0.04, 0.2	0, 1, 0
VMT-TRE	0.03, 0.9, 0	0.9, 0.4, 0.1	0.2, 0.6, 0.01
PBS	23.4, 0, 0.6	0.3, 0.6, 0.01	12.3, 0.03, 0.4
GMFM-stand	8.6, 0, 0.3	0, 1, 0	1.5, 0.2, 0.1
GMFM-walk	9.3, 0, 0.3	0.1, 0.8, 0	3.8, 0.1, 0.2

(TPL is total path length, SR is success rate, and TRE is total residual error.)

**Table 7:** Results of paired t-test of comparison between pre- and post-intervention COP-TPL. Presented are group, means and standard errors of means (SEM), t-statistic, *p*-value, and effect size.

	Mean (SEM)		t-Statistic	*p*-Value	Effect Size
	Pre	Post			
XG					
EO	50.9 (5.8)	39.3 (5.7)	2.52	0.03	0.8
EC	57.7 (7.3)	48.3 (5.4)	1.68	0.1	0.5
VMT	59.9 (8.7)	45.4 (5.4)	2.4	0.05	0.1
VCG	64.6 (5.8)	46.3 (6.6)	4.23	0.001	1.3
CG					
EO	38.7 (5.3)	44.6 (5.5)	−1.15	0.3	0.3
EC	39.1 (4.5)	42.7 (4.2)	−1.96	0.1	0.6
VMT	36.8 (3.9)	40.6 (4.6)	−1.01	0.3	0.3
VCG	51.9 (6.7)	44.1 (4.8)	2.35	0.04	0.7

(EO is eyes open, EC is eyes closed, VMT is visuomotor tracking task, and VCG is visual cognitive game.)

## Data Availability

On request the data is available with the corresponding author.

## Appendix A

**Table A1:** List of computer video games used in this study, obtained from www.bigfishgames.com (15 November 2021). Note that matching and shooting games require children to use a small wireless hand-held clicker with a left mouse button to press when needed for game play.

Big Fish Game	Axis Play	Start Difficulty	Type	Clicker	Precision	Background	Distractor	Executive Function
Abundante	Horizontal	Difficult	Match 3	Yes	Moderate	Low optokinetic	No	Matching and puzzle solving
Action ball	Horizontal	Moderate	Brick Buster	No	Moderate	High optokinetic	Yes	Visual tracking and spatial
Acqua Ball	Horizontal	Easy	Brick Buster	No	Low	Medium optokinetic	Yes	Visual tracking and spatial
Astrobugs Revenge	Horizontal	Difficult	Match 3	Yes	High	Medium optokinetic	No	Matching
Ark Light	Variable	Moderate	Shooting	Yes	Moderate	Medium optokinetic	Yes	Search and select
Birds Town	Horizontal	Moderate	Match 3	Yes	High	Low optokinetic	No	Matching
Brave Piglet	Vertical	Moderate	Shooting	Yes	High	Low optokinetic	Yes	Visual tracking and spatial
Bricks of Egypt	Horizontal	Easy	Brick Buster	No	Variable	Low optokinetic	Yes	Visual tracking and spatial
Butterfly Escape	Horizontal	Moderate	Match 3	Yes	High	Low optokinetic	Yes	Visual tracking and spatial
Chicken Invaders	Variable	High	Shooting	Yes	Moderate	High optokinetic	Yes	Search and select
Digby Donuts	Horizontal	Moderate	Catch and Sort	Yes	Moderate	Low optokinetic	No	Search precision sort
Feeding Frenzy	Variable	Difficult	Aim and Move	No	High	Low optokinetic	Yes	Visual tracking and spatial
Invadazoid	Horizontal	Difficult	Brick Buster	No	High	Moderate optokinetic	Yes	Visual tracking and spatial
Jar of Marbles	Horizontal	Easy	Match 3	Yes	Medium	Low optokinetic	No	Matching three, aligning
Jet Jumper	Variable	Difficult	Driving Game	Yes	High	High optokinetic	Yes	Visual tracking and driving
Luxor 3	Horizontal	Moderate	Match 3	Yes	High	Moderate optokinetic	Yes	Match 3, aligning
Luxor HD	Horizontal	Moderate	Match 3	Yes	High	Moderate optokinetic	Yes	Match 3, aligning
Reaxion	Horizontal	Moderate	Brick Buster	No	Variable	Moderate optokinetic	Yes	Visual tracking and spatial
Ricochet Recharge	Horizontal	Moderate	Brick Buster	No	High	Moderate optokinetic	No	Visual tracking and spatial
